# Interactions Between Blood Nutritional Biomarkers and Apolipoprotein E ε4 in the Progression of Mild Cognitive Impairment in Alzheimer’s Disease

**DOI:** 10.3390/nu18081263

**Published:** 2026-04-16

**Authors:** Rasheedat Lawal, Sanjay Kumar, Rosemary Chigevenga, Shelly Coe

**Affiliations:** 1 School of Sport, Nutrition and Allied Health Professions, Oxford Brookes University, Oxford OX3 0BP, UK; scoe@brookes.ac.uk; 2 School of Psychology, Social Work and Public Heath, Oxford Brookes University, Oxford OX3 0BP, UK; skumar@brookes.ac.uk (S.K.); rchigevenga@brookes.ac.uk (R.C.)

**Keywords:** mild cognitive impairment (MCI), Alzheimer’s disease, nutritional biomarkers, blood nutritional biomarkers, genetic risk factors, apolipoprotein E, dementia

## Abstract

Background/Objectives: Mild cognitive impairment (MCI), the prodromal stage of Alzheimer’s disease, may be influenced by nutritional status and genetic susceptibility. This systematic review synthesised evidence on how nutritional biomarkers interact with genetic variants, particularly APOE ε4, to influence cognitive outcomes in individuals with MCI. Methods: Following PRISMA 2020 guidelines, seven studies were included (three longitudinal, two randomised controlled trials, and two cross-sectional) involving adults aged ≥55 years with MCI. Nutritional exposures comprised plasma or serum concentrations of vitamins A, D, E, the vitamin B group, lipids, selenium, and ketogenic medium-chain triglycerides. Genetic risk was assessed primarily through APOE ε4 status. Risk of bias was assessed using RoB 2 and ROBINS-I, and certainty of evidence using GRADE. Due to heterogeneity in biomarkers, cognitive tools, and study designs, findings were synthesised narratively. Results: Across nutrient categories, higher concentrations of vitamin D, selenium, and antioxidants were associated with better cognitive outcomes. kMCT supplementation improved episodic memory and brain energy metabolism. Evidence for nutrient–gene interactions was mixed: APOE ε4 modified responses to vitamin B group and selenium but showed limited influence on vitamin D, lipids, or kMCT effects. Heterogeneity in biomarker assays, cognitive tools, and genetic stratification limited comparability across studies. Conclusions: Nutritional biomarkers appear to influence cognitive trajectories in MCI, and some associations may differ by APOE ε4 status. However, small samples and limited genetic stratification constrain interpretation. Future research should prioritise standardised biomarker measurement, genetically stratified cohorts, and individual participant data meta-analyses to clarify nutrient–gene interactions in MCI.

## 1. Introduction

Alzheimer’s disease (AD) is the most common cause of dementia [1], which accounts for 55.2 million dementia cases estimated globally and is projected to exceed 75.6 million cases by 2030 [2]. In the United Kingdom, nearly 1 million individuals are currently affected, with numbers expected to reach 1.4 million by 2040 [3,4].

The exact aetiology of AD remains incompletely understood, despite extensive research efforts [2,5,6]. Genetic factors account for about 60–80% of the development of AD [2,7,8], with genetic materials containing polygenic genes that are either inherited as mutated genes or sporadic in nature [2,5,9]. In the late-onset groups, the apolipoprotein E (APOE) gene, the strongest and most consistently replicated genetic risk factor, is associated with AD. The ε4 allele increases the likelihood of developing AD and is associated with greater neuroinflammation and impaired lipid transport [10]. In contrast, the young onset familial AD, representing only 1–6% of cases, is driven by rare autosomal-dominant mutations in amyloid precursor protein (APP), presenilin 1 (PSEN1) and presenilin 2 (PSEN2) [2,5,11]. These deterministic variants typically present before age 65 [5,9,12] and follow distinct clinical and neuropathological trajectories. The Primary Care Dementia Data from August 2025 reported that 6.8% of the recorded diagnoses of dementia were diagnosed between 40 and 64 years of age [13]. 

Mild cognitive impairment (MCI) is widely recognised as the prodromal stage of Alzheimer’s disease. It is characterised by progressive loss of memory, thinking, and cognitive abilities, including language and attention [14], between normal ageing and Alzheimer’s disease, but does not interfere notably with daily living and activities [15] and has become an important indicator for the preliminary stages of dementia.

Although individuals with MCI face an elevated risk of progression to Alzheimer’s disease [2,16,17], if not controlled properly [18], the trajectories are highly variable. Longitudinal studies indicate that approximately 30% of individuals with MCI remain stable over time [14], and up to 26% may revert to normal cognition [19]. This suggests that MCI represents a critical window of opportunity during which modifiable risk factors, including nutritional status, may help stabilise cognition or delay the progression to AD [18].

Growing evidence suggests that nutritional status plays a key role in maintaining cognitive function and may influence the risk of progression from MCI to AD [16,18,20,21,22]. Several nutrients, including the vitamin B group, vitamin D, choline, antioxidants, and selenium, contain neuroregenerative and neuroprotective properties and are essential for neurotransmitter synthesis, are beneficial for brain health, are protective against oxidative stress and damage, and they promote the growth of nerve cells [20,22,23]. Deficiencies in these nutrients have been associated with structural brain changes and accelerated cognitive decline [20,22].

Observational studies have consistently reported lower circulating levels of key nutrients in individuals with MCI compared with cognitively healthy adults. For example, van Wijk et al. [22] found reduced blood concentrations of folate, uridine, and choline, and elevated homocysteine in MCI and AD, suggesting metabolic vulnerability. Baumel et al. [20] similarly reported that the limited availability of choline, docosahexaenoic acid (DHA), and uridine may compromise phospholipid synthesis and neuronal repair. The vitamin B group, particularly folate and vitamin B12, is central to folate-dependent metabolic pathways, as deficiencies lead to elevated homocysteine, a neurotoxic amino acid linked to oxidative stress, vascular dysfunction, and increased risk of AD [24,25].

Vitamin D has also been associated with cognitive health via its roles in immune regulation. Several studies have reported associations between low serum 25-hydroxyvitamin D (250HD) and poorer cognitive performance or increased risk of cognitive decline [26]. Antioxidant vitamins A and E protect neuronal membranes from lipid peroxidation, and deficiencies have been associated with significant cognitive impairment [27]. Also, selenium, an essential trace element involved in enzyme systems, has been linked to cognitive decline, although findings remain inconclusive [28].

Beyond observational associations, interventional studies have explored whether nutritional supplementation can improve cognitive outcomes in MCI. Fortier et al. [29] demonstrated that ketogenic medium-chain triglyceride (kMCT) supplementation increases plasma ketone availability, enhances brain energy metabolism, and improves episodic memory, an early domain affected in AD. Other trials have examined combinations of vitamin B groups or multi-nutrient formulations, with varying degrees of success. However, heterogeneity in study design, nutrient dosage, biomarker measurement, and cognitive assessment tools complicates comparisons across studies.

Blood-based nutritional biomarkers offer an objective means of assessing nutrient status and may provide more reliable indicators of physiological availability than dietary intake measures, which are prone to recall bias and measurement error [22]. Biomarkers also allow for the investigation of nutrient–gene interactions, which may help explain individual variability in cognitive responses to nutritional exposures.

Emerging studies suggest that APOE ε4 may modify the relationship between nutritional status and cognitive outcomes. For example, Shahr et al. [27] reported that APOE ε4 carriers exhibited lower serum vitamin E concentrations and greater cognitive impairment, suggesting increased vulnerability to oxidative stress. Gordon et al. [30] found that low vitamin B status and elevated homocysteine were associated with a substantially higher risk of progression from MCI to AD, with APOE ε4 carriers showing increased risk. Selenium is suggested to confer cognitive benefits primarily in APOE-negative individuals, with weaker associations observed in carriers [28]. On the other hand, APOE ε4 showed limited influence on vitamin D cognition associations and inconsistent effects on lipid-related cognitive outcomes [10,26]. Findings from kMCT supplementation studies also suggest that APOE ε4 may reduce responsiveness to ketone-based interventions [29,31].

Despite these insights, the evidence base remains fragmented. Most studies investigate single nutrients in isolation, use small samples, or lack sufficient genetic stratification. Several gaps justify the need for this review. Although numerous studies examine nutritional biomarkers of APOE ε4 independently, they investigate their interaction in AD but not in MCI. Also, most studies focus exclusively on APOE ε4, with little inclusion of other genetic variants.

This systematic review, therefore, synthesises evidence on the interactions between blood nutritional biomarkers/dietary supplementation and APOE ε4 status in individuals with MCI. It aims to determine whether nutritional and genetic risk factors interact to influence cognitive outcomes in the progression of MCI to Alzheimer’s disease.

## 2. Materials and Methods

### 2.1. Study Design and Registration

The protocol for this systematic review was registered in the International Prospective Register of Systematic Reviews (PROSPERO; CRD420251126402) and adhered to the Preferred Reporting Items for Systematic Reviews and Meta-Analyses (PRISMA) 2020 [32] guidelines for reporting. The completed PRISMA 2020 Checklist is available in Supplementary File S1.

This study used a systematic review to investigate the role of nutritional factors in the progression of mild cognitive impairment to Alzheimer’s disease, with a focus on interactions with genetic variants.

### 2.2. Eligibility Criteria

Studies were eligible if they were cross-sectional, longitudinal, or randomised controlled trials (RCTs) involving adults diagnosed with mild cognitive impairment (MCI). Both young-onset (45–64 years) and late-onset (≥65 years) MCI populations were included. Eligible studies examined either (i) blood-based nutritional biomarkers or (ii) dietary or nutrient-based interventions aimed at supporting cognitive function in individuals with MCI.

To meet the review objective, studies were required to assess at least one genetic variant associated with Alzheimer’s disease risk, primarily APOE ε4, but also APP, PSEN1, or PSEN2 where available, and to report cognitive outcomes using validated tools. Nutritional exposures included plasma or serum concentrations of nutrients such as vitamin D, vitamin B group, selenium, vitamins A and E, lipids, or ketone-based metabolites, as well as supplementation with nutrients relevant to cognitive health.

Studies were excluded if they were reviews, editorials, conference abstracts, animal studies, or involved participants without MCI (e.g., cognitively healthy adults or individuals with dementia). Studies involving participants with other chronic medical conditions were also excluded to reduce confounding.

Only studies with eligible outcomes were categorised based on individuals with a clinical diagnosis of MCI and provided evidence of diagnostic criteria that were valid for assessment using the Mini-Mental State Examination (MMSE) and Montreal Cognitive Assessment (MoCA) as tools for assessing cognitive.

### 2.3. Information Sources and Search Strategy

Potentially relevant studies were identified through a comprehensive search of electronic databases (Academic Search Complete, MedLine, National Library of Medicine (NLM), Web of Science and Research and Digital Assets Repository (RADAR)) to identify studies between 2010 and 2025. The search through databases was conducted in August 2025 to identify journals reporting on nutritional biomarkers or interventions aimed at improving cognition in MCI and investigating their interactions with genetic variants responsible for the progression of AD in MCI.

Search terms were identified by using the PICO framework to include individuals with MCI and genetic variants responsible for AD (population), nutritional/dietary interventions, nutritional biomarkers and the use of supplements including nutrients/micronutrients responsible for improving cognition (intervention), comparing carriers versus non-carriers of genetic variants (comparator), to slow the risk of progression to developing AD (outcome), using Medical Subject Headings (MeSH) terms and combinations of keywords. The inclusion and exclusion criteria are outlined in Table 1.

Used search terms and keywords included relevant words that were related to nutrition/diet/biomarkers, neurocognitive terms, and genetic factors/variants, with Boolean operators “OR” and “AND” used to combine them to further define the search strategy and identify relevant studies. A full list of keywords and MeSH terms used has been provided in Appendix A. Search terms used were determined with consultations with a librarian. The reference lists of identified relevant articles were searched manually and included for screening. The search strategy was limited to studies in the English language only. All citations were imported into EndNote Library [33] to remove duplicates.

### 2.4. Selection and Data Collection Process

The selection process followed the PRISMA 2020 [32] guidelines. Titles and abstracts of identified and retrieved records were independently screened by one researcher/reviewer, and 10–20% were checked by a second reviewer for relevance based on the predefined inclusion and exclusion criteria. The flow chart describing the process of the study selection using the PRISMA 2020 guidelines is represented in the Results.

The full text of articles that met the inclusion criteria was then assessed independently by the same researcher/reviewer and checked independently by a second assessor. If any conflicts arise between the two assessors, a third assessor reviews the paper. EndNote Library [34] was used to search for the full text of articles. Where titles and abstracts met the inclusion criteria but did not contain the full text, the reviewer reached out to university librarians for the full text of articles or requested it from original authors via ResearchGate. Any uncertainties during the selection process were resolved by consulting the relevant literature or through discussions within the research team.

### 2.5. Data Items

For each included study, the following data were extracted:

- Characteristics of the Study: First author/year of publication, study design/duration, objective of the study, study population, and sample size.
- Participant Demographics: Age and diagnosis.
- Exposure: type of intervention.
- Measures: samples collected, assessments/questionnaires administered, and other covariates used for analysis relevant to diet, cognitive function and genetic variants.

Exposures of interest included intake of daily dietary supplements, plasma/serum nutrient concentration, standardised cognitive assessments (Mini-Mental State Examination, Montreal Cognitive Assessment), and blood samples for genetic testing to determine the presence of APOE ε4.

### 2.6. Study Risk of Bias Assessment

Risk of bias was assessed for each study included using the appropriate validated tools for the study design. Randomised controlled trials were evaluated using the Cochrane Risk of Bias (RoB 2) tool [35] across five domains. Longitudinal and cross-sectional studies were assessed using the Risk of Bias in Non-randomised Studies of Interventions (ROBINS-I) [36].

All assessments were conducted independently by one reviewer, with 10–20% cross-checked by a second reviewer. To enhance consistency and minimise subjective bias, a pilot screening of a random sample of records was conducted to refine the criteria. Records were screened using predefined eligibility criteria and refined where necessary to improve clarity and reduce subjectivity.

### 2.7. Effect Measures

Cognitive outcomes were assessed using MMSE in six studies and MoCA in one study. Because these instruments differ in scoring range and psychometric properties, raw cognitive scores were not pooled across studies. Instead, cognitive findings were synthesised narratively, with results reported separately for each study.

Effect measures were not synthesised meta-analytically due to heterogeneity in study design and biomarker units. No transformations or pooled analyses were conducted on nutrient concentrations. Where available, associations between nutrient levels and cognitive outcomes were summarised descriptively.

### 2.8. Synthesis Methods

Given the heterogeneity in study designs, biomarker assays, nutrient categories, and cognitive assessments, a narrative synthesis approach was used. Studies were grouped thematically by nutrient category (vitamin D, kMCTs, vitamin B group, selenium, lipids, vitamins A and E), and findings were summarised in relation to cognitive outcomes and APOE ε4 status.

No statistical pooling, regression modelling, or correlation analysis was performed. Instead, patterns of association were described qualitatively, with attention to consistency, directionality, and the presence or absence of nutrient–gene interactions. Risk of bias assessments informed the interpretation of findings but were not used to weight studies quantitatively.

### 2.9. Reporting Bias Assessment

Reporting bias was not assessed using formal quantitative methods because no meta-analysis was conducted, and effect sizes were not pooled across studies. Funnel plots and statistical tests for small study effects were therefore not applicable.

Instead, potential reporting bias was considered narratively. We examined whether there was evidence of selective outcome reporting, discrepancies between prespecified and reported outcomes (where protocols were available), and inconsistencies between described methods and presented results. Selective reporting of cognitive outcomes, nutrient biomarkers, and APOE ε4 stratification was evaluated using the relevant domains of the Cochrane Risk of Bias 2 (RoB 2) [35] and the Risk of Bias in Non-randomised Studies of Interventions (ROBINS-I) [36] tools.

### 2.10. Certainty Assessment

Certainty of evidence was evaluated using the GRADE framework, adapted for a narrative synthesis without meta-analysis. Certainty ratings were applied qualitatively based on the available evidence rather than pooled effect estimates.

It was evaluated across five domains applied across plasma or serum containing vitamins, nutrients, and ketogenic medium-chain triglycerides (kMCTs) with APOE stratification and assessed the certainty of evidence as moderate, low to moderate, and low using the recommendations described in the Cochrane Handbook for Systematic Reviews of Interventions. Each exposure–outcome pair was evaluated across five domains: risk of bias, inconsistency, indirectness, imprecision, and publication bias.

## 3. Results

### 3.1. Study Selection

**Figure 1:**
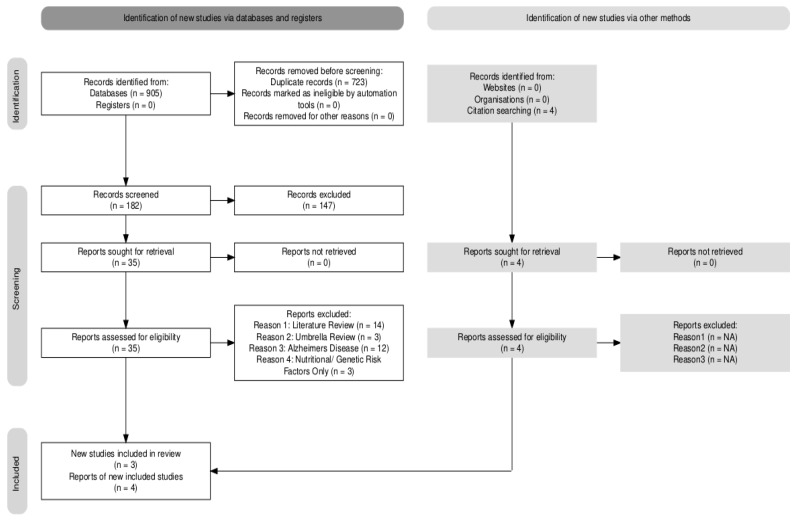
PRISMA flow diagram. Abbreviations: NA (Not Applicable).

A total of 909 records were identified through three electronic database searches and citation searches. After removing 723 duplicates, 182 records were screened by title and abstract, 39 full-text articles were assessed, and 7 studies met the inclusion criteria: 3 longitudinal studies, 2 randomised controlled trials, and 2 cross-sectional studies. These studies investigated nutritional exposures (concentration of vitamins in plasma or serum and dietary supplementation) and genetic variants in individuals with mild cognitive impairment. A PRISMA flow diagram is presented in Figure 1.

### 3.2. Study Characteristics

A total of seven studies met the inclusion criteria. This comprised three longitudinal studies, two randomised controlled trials, and two cross-sectional studies with sample sizes ranging from 19 to 4553 participants. All studies collectively investigated the association of nutritional exposures in serum/plasma in individuals with mild cognitive impairment compared to cognitively healthy individuals with stratification by APOE ε4 carrier status.

Nutritional exposures included serum/plasma containing vitamins A, D, and E; vitamin B group; selenium; lipids and ketogenic medium-chain triglycerides (kMCTs); dietary supplementation of kMCTs; and genetic variants (primarily APOE ε4) in relation to cognitive decline in mild cognitive impairment. Cognitive outcomes were assessed using the Mini-Mental State Examination (MMSE) in six studies and the Montreal Cognitive Assessment (MoCA) in one study.

Table 2 below summarises key characteristics of the individual studies.

### 3.3. Risk of Bias in Studies

Risk of bias in the studies was assessed using two Cochrane-approved tools tailored to study designs. Each study was independently evaluated, and discrepancies were resolved through discussion.

Most studies demonstrated low risk in outcome measurement and confounding control. One study showed a high risk due to limited adjustment and potential selective reporting. No studies were excluded based on risk of bias, and because no meta-analysis was conducted, no sensitivity analyses were required. Instead, risk of bias assessments informed the qualitative interpretation of findings.

### 3.4. Results of Individual Studies

Seven studies included in this review individually explored the interaction between nutritional exposures and genetic risk factors in individuals between the ages of 55 and 90 years diagnosed with MCI and carriers of the APOE ε4 gene.

Findings are presented thematically by nutrient category, integrating both blood biomarkers and dietary supplement intake where applicable.

#### 3.4.1. Serum Containing Vitamin D

Dursun et al. [26] investigated the level of key nutrients, particularly 25-hydroxyvitamin-D3 (25OHD), the major form of vitamin D, which is beneficial to brain health, in the serum (the protein-rich liquid component of the blood that provides immunity to the development of diseases). They reported that higher serum 25-hydroxyvitamin D (25OHD) concentrations were associated with better MMSE performance in individuals with MCI. Although no significant deficiency was observed at baseline, higher 25OHD levels predicted more favourable cognitive trajectories. Among APOE ε4 carriers, elevated 25OHD levels were associated with higher cognitive scores, suggesting a potential genotype-modulated relationship between vitamin D status and cognitive resilience.

#### 3.4.2. Ketogenic Medium-Chain Triglycerides (kMCTs)

Studies by Fortier et al. [29] and Fortier et al. [31] sought to provide evidence that supplements containing kMCTs, a type of MCT, may help to address brain energy deficits and serve as an alternative source of energy to the brain to improve cognition in individuals with MCI. Dietary intake of kMCTs was assessed through daily supplementation, and plasma samples were collected to evaluate metabolic responses to the intervention. This demonstrated improvements in cognition (episodic memory and visual memory) evident in cognitive assessments and daily functioning, with an increase in brain ketone uptake and brain energy supply. Furthermore, Fortier et al. [31] suggested that APOE ε4 may impede ketone utilisation, although larger samples are needed to confirm this interaction.

#### 3.4.3. Vitamin B Group

Gordon et al. [30] reported that lower serum folate and vitamin B12 levels were associated with elevated homocysteine and poorer cognitive outcomes. APOE ε4 carriers with elevated homocysteine showed the most pronounced cognitive decline, suggesting a potential nutrient–gene interaction.

#### 3.4.4. Serum Selenium

Kim et al. [28] found that lower serum selenium concentrations were associated with poorer cognitive performance and faster decline. The association between selenium and cognition was stronger in APOE ε4 carriers, indicating possible genotype-specific vulnerability to oxidative stress.

#### 3.4.5. Lipid Profiles

Ren et al. [10] reported that elevated LDL cholesterol and triglycerides were associated with accelerated cognitive decline, with APOE ε4 carriers demonstrating more pronounced lipid-related cognitive decline, consistent with APOE’s role in lipid transport and metabolism.

#### 3.4.6. Antioxidant Vitamins A and E

Shahar et al. [27] found that lower serum vitamin A and E levels were associated with poorer cognitive performance. Subsequently, APOE ε4 carriers with low antioxidant status showed the greatest cognitive impairment, suggesting heightened oxidative vulnerability.

### 3.5. Results of Synthesis

The seven included studies were synthesised narratively due to heterogeneity in study design, nutritional exposures, and cognitive outcome measures. Findings were grouped thematically by nutrient category (vitamin B group, antioxidant vitamins A and E, vitamin D, selenium, lipids, and ketogenic medium-chain triglycerides (kMCTs)) and summarised according to direction and consistency of reported associations.

Across studies, higher blood concentrations of vitamin D, selenium, and antioxidant vitamins were associated with better cognitive outcomes, whereas elevated homocysteine, adverse lipid profiles, and low antioxidant status were associated with poorer cognition. Also, dietary intake findings complemented biomarker evidence (kMCT supplementation was associated with improvements in cognitive performance). APOE ε4 status consistently modified these associations, with carriers showing either weakened benefits (e.g., kMCTs) or more pronounced adverse effects (e.g., elevated homocysteine).

Although the studies varied in design, nutritional exposures, and cognitive measures, the overall pattern across studies indicated that nutritional status and dietary composition were linked to cognitive trajectories in MCI and that APOE ε4 carriers often demonstrated differential responses to nutritional exposures.

### 3.6. Reporting Biases

Reporting bias was assessed qualitatively because a meta-analysis was not conducted. Across the included studies, selective reporting was judged to be a moderate concern.

**Figure 2:**
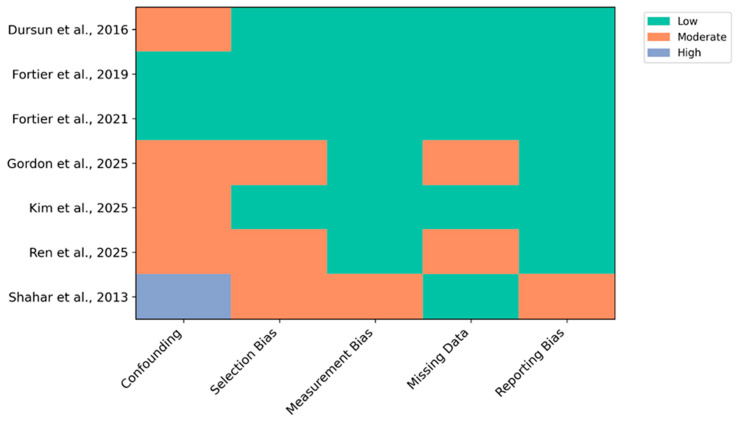
Risk of bias plot [10,26,27,28,29,30,31].

Randomised controlled trials [29,31] showed consistently low risk across domains, strengthening confidence in their findings. Cross-sectional and longitudinal [10,30] had moderate risk due to potential confounding and incomplete follow-up, with Shahar et al. [27] showing high risk due to limited adjustment and potential selective reporting. A risk of bias plot is illustrated in Figure 2.

### 3.7. Certainty of Evidence

Certainty of evidence was assessed using an adapted GRADE approach suitable for narrative synthesis. Certainty ratings varied across nutrient categories and exposure outcome pairs. Evidence for vitamin D and kMCT supplementation was rated as moderate, supported by consistent findings across multiple studies with validated cognitive assessments. Evidence relating to selenium, antioxidant vitamins (A and E), and lipid biomarkers was rated as low to moderate, reflecting variability in biomarker measurement and limited genotype-stratified analyses. Evidence for the vitamin B group is low, primarily due to reliance on a single study and potential confounding.

Across all categories, certainty was reduced by heterogeneity in study design, variation in dietary assessment methods, and limited representation of young-onset MCI. Certainty was highest in studies that stratified results by APOE ε4 status and used longitudinal follow-up with validated cognitive measures.

## 4. Discussion

This systematic review synthesised evidence from seven studies examining the relationship between blood nutritional biomarkers/dietary supplementation and APOE ε4 status in individuals with MCI. Across nutrient categories, two consistent themes emerged: nutritional status is associated with cognitive trajectories in MCI, and APOE ε4 carriers often exhibit differential or diminished responses to nutritional exposures.

Higher blood concentrations of vitamin D, selenium, and antioxidant vitamins A and E were generally associated with more favourable cognitive outcomes. These nutrients are implicated in neuroprotection through antioxidant, anti-inflammatory, and metabolic pathways [27], suggesting that adequate nutritional status may support cognitive stability in MCI. Conversely, elevated homocysteine, adverse lipid profiles, and low antioxidant status were associated with poorer cognitive performance. The relationship between vitamin B status and homocysteine is well established, as folate and vitamin B12 are required for folate-dependent metabolic pathways, and insufficient availability leads to elevated plasma homocysteine, a marker linked to oxidative stress [24], and increased risk of AD [37]. The findings from Gordon et al. [30] align with this biological framework, although evidence remains limited to a single longitudinal study.

Dietary intake findings complemented biomarker evidence. kMCT supplementation was associated with improvements in cognitive performance, consistent with the role of ketone bodies as an alternative energy source in the context of impaired cerebral glucose metabolism [31,38].

Across nutrient categories, APOE ε4 status consistently modified associations between nutritional exposures and cognitive outcomes. Carriers often exhibited either attenuated benefits, such as reduced responsiveness to kMCT supplementation, or more pronounced adverse effects, including stronger associations between elevated homocysteine and cognitive decline. For example, Gordon et al. [30] found that low plasma levels of vitamin B_12_, B_6,_ and riboflavin, as well as elevated homocysteine, were independently associated with increased risk of progression of AD, regardless of APOE status.

These patterns are consistent with APOE ε4’s established roles in lipid transport and neuroinflammation, suggesting that genetically vulnerable individuals may be more sensitive to nutritional imbalances or metabolic stressors. Although the evidence base remains limited, these findings highlight the potential value of genotype-stratified approaches in nutritional research and intervention design.

Notably, other genetic variants such as APP, PSEN1, and PSEN2 are associated with young-onset MCI [2,12] were not represented in the included studies. This limits the generalisability of findings across age groups and genetic subtypes. Furthermore, comparisons between young-onset and late-onset MCI are scarce, despite evidence that young-onset cases may present with distinct neuropsychological profiles and faster progression rates [6,14]. These gaps limit our understanding of whether nutritional exposures are equally relevant across age groups and genetic backgrounds.

These findings highlight the need for larger, long-term genotype-stratified trials to clarify whether nutritional interventions exert differential effects across APOE ε4 carriers and non-carriers.

The interaction between nutritional exposures and genetic susceptibility remains underexplored. Also, small sample sizes, short follow-up durations, and inconsistent reporting of genetic stratification hinder definitive conclusions. Emerging research in nutrigenomics, the interaction between diet and genes in the progression of diseases, suggests that single-nucleotide polymorphisms (SNPs) and polygenic risk scores (PRSs) may influence nutrient metabolism, inflammation, and cognitive resilience [2,5].

### Strengths and Limitations

Strengths of this review include adherence to PRISMA 2020 guidelines, the use of validated cognitive assessments across studies, and the inclusion of both biomarker and dietary intake data. However, several limitations must be acknowledged. The evidence base is small, with some nutrient categories represented by only one study. Heterogeneity in study design, dietary assessment methods, biomarker thresholds, and follow-up duration limits comparability. Sample sizes were small in several studies, and young-onset MCI populations were under-represented. Observational designs also introduce the possibility of residual confounding, particularly in studies examining the vitamin B group, homocysteine, and lipid biomarkers.

Studies focused on APOE ε4, which is the most common genetic risk factor for Alzheimer’s disease and consistently examined across the available literature on nutrition, MCI, and AD risk [39,40]. Other AD-related variants (APP, PSEN1, and PSEN2) were not represented in the included data, as these mutations are extremely rare and primarily associated with autosomal-dominant, young-onset AD. Research involving these deterministic mutations typically requires specialised genetic counselling and family-based recruitment, which rarely overlaps with nutritional biomarker or lifestyle-based study designs [41]. As a result, the generalisability of findings to young-onset genetically at-risk individuals or rare forms of MCI remains limited. Future studies should consider broader genetic stratification and the inclusion of young-onset MCI populations to explore potential age and genotype-related differences in nutritional responses.

## 5. Conclusions

This systematic review highlights consistent associations between nutritional status, APOE ε4 genotype, and cognitive trajectories in MCI. Although evidence remains limited, emerging patterns suggest that genetic susceptibility may influence responsiveness to nutritional exposures. Larger, genotype-stratified studies are needed to clarify these interactions and inform more personalised approaches to nutritional risk reduction in cognitive decline.

## Figures and Tables

**Table 1:** Inclusion and exclusion criteria based on PICO framework.

PICO Element	Inclusion Criteria	Exclusion Criteria
Population	Individuals clinically diagnosed with MCI aged 45–64 years (young onset) and ≥65 years (late onset).	Individuals with normal cognition or AD. Individuals with chronic conditions.
Intervention/Exposure	Plasma/Serum Biomarkers. Dietary/Nutritional Interventions.	Studies with no nutrient or genetic exposure measured.
Comparator	Carriers versus non-carriers of APOE, APP, PSEN1, and PSEN2 genetic variants.	No comparator group.
Outcome	Cognitive outcomes using validated tools. Risk of progression to AD.	Studies without cognitive outcomes.

Abbreviations: MCI (mild cognitive impairment), AD (Alzheimer’s disease), APOE (apolipoprotein E), APP (amyloid precursor protein), PSEN1 (presenilin 1), PSEN2 (presenilin 2).

**Table 2:** Summary of studies on nutritional factors, genetic risk factors, and cognition.

Study (Year)	Design	Objective	Population (*n*)	Exposure and Measures	Key Findings
Dursun et al. [26]	Longitudinal study, 6-year duration	Investigate serum levels of 25OHD in MCI and its correlation with APOE ε4	MCI 60–78 years (*n* = 32)	Serum Vitamin D APOE status MMSE	Higher 25OHD levels are associated with better cognition; APOE ε4 carriers had higher 25OHD but no cognitive difference
Fortier et al. [29]	RCTs, 6-month duration	Determine improvements to cognition with kMCTs in older people	MCI ≥55 years (*n* = 19)	Plasma kMCTs Dietary supplementation of kMCTs MMSE Blood testing APOE status	Improved brain energy and episodic memory in all participants, but no APOE ε4 effect
Fortier et al. [31]	RCTs, 6-month duration	Determine improvements to cognition with kMCTs in older people	MCI ≥55 years (*n* = 39)	Plasma kMCTs Dietary supplementation of kMCTs MMSE Blood testing APOE status	Cognitive improvement: APOE ε4 may reduce responsiveness to ketones
Gordon et al. [30]	Longitudinal Study, 4-year duration	Determine the interaction between the APOE ε4 and the vitamin B group in cognitive outcomes	MCI ≥60 years (*n* = 4553)	Serum folate, vitamins B_12_, B_6_ and riboflavin Plasma homocysteine MMSE Blood testing	Low B-vitamin status and high homocysteine linked to increased risk; APOE ε4 amplified risk
Kim et al. [28]	Longitudinal Study, 2-year duration	Examine the relationship between serum Se levels and cognition with APOE ε4	MCI 60–90 years (*n* = 113)	Serum Se Blood testing APOE status	Selenium protective in APOE-negative MCI; weaker effect in APOE ε4 carriers
Ren et al. [10]	Cross-sectional study	Investigate the relationship between plasma lipid levels, cognition and genetic variants	MCI ≥73 years (*n* = 177)	MoCA Blood testing APOE status	Lipid-cognition associations are strongest in low polygenic risk; APOE influence unclear
Shahar et al. [27]	Cross-sectional study	Test the effect of APOE ε4 on serum concentration of vitamins A and E in MCI	MCI ≥60 years (*n* = 67)	Serum vitamins A and E MMSE Blood testing	Vitamin A and E deficiency linked to MCI progression; APOE ε4 associated with lower serum vitamin A and E

Abbreviations: RCTs (randomised controlled trials), MCI (mild cognitive impairment), MMSE (Mini-Mental State Examination), MoCA (Montreal Cognitive Assessment), APOE ε4 (apolipoprotein E ε4), 25OHD (25-hydroxyvitamin D), kMCT (ketogenic medium-chain triglyceride), Se (selenium).

## Data Availability

The corresponding author, R.L. (Rasheedat Lawal), will provide any additional data related to this study upon reasonable request.

## Supplementary Materials

The following supporting information can be downloaded at: https://www.mdpi.com/article/10.3390/nu18081263/s1, Supplementary File S1: PRISMA 2020 Checklist.

## Abbreviations

The following abbreviations are used in this manuscript:

MCI	Mild Cognitive Impairment
APOE ε4	Apolipoprotein E ε4 Allele
APP	Amyloid Precursor Protein
PSEN1	Presenilin 1
PSEN2	Presenilin 2
RCTs	Randomised Controlled Trials
kMCTs	Ketogenic Medium-Chain Triglycerides
MCT	Medium-Chain Triglycerides
LDL	Low-Density Lipoprotein
AD	Alzheimer’s Disease

## Appendix A

Search Strategy Terms and Keywords
  Nutritional/Dietary Terms: “Diet*”, “Nutrition”, “Nutritional Factor*”, “Dietary Intervention*”, “Nutritional Intervention*”, “Nutritional Biomarker*”, “MIND Diet*”, “Micronutrient*”, “Supplement*”
  Neurocognitive Terms: “Mild Cognitive Impairment”, “Cognitive Impairment”, “Cognitive Decline”, “Cognitive Function*”, “Alzheimer’s Disease”, “Alzheimer*”
  Genetic Terms: “Genetic Risk*”, “Genetic Risk Factor*”, “Genetic Variant*”
  Boolean Operators
  “AND” used to combine PICO elements
  “OR” used to expand within concept groups
